# Primary care physicians working in rural areas provide a broader scope of practice: a cross-sectional study

**DOI:** 10.1186/s12875-023-02250-y

**Published:** 2024-01-02

**Authors:** Makoto Kaneko, Tomoya Higuchi, Ryuichi Ohta

**Affiliations:** 1 Department of Health Data Science, Yokohama City University, 22-2, Seto, Kanazawa-Ku, Yokohama, Kanagawa 236-0027 Japan; 2 Department of Family and Community Medicine, Hamamatsu University School of Medicine, 1-20-1, Handayama, Higashi-Ku, Hamamatsu, 431-3192 Japan; 3 Shizuoka Family Medicine Program, 1-20-1, Handayama, Higashi-Ku, Hamamatsu, 431-3192 Japan; 4 Department of Community Care, Unnan City Hospital, 96-1, Iida, Daito-Cho, Unnan, Shimane 699-1221 Japan

**Keywords:** Primary care, Scope of practice, Rurality, Japan

## Abstract

**Background:**

Scope of practice (SoP) is an important factor for primary care physicians (PCPs). One of the strong determinants of SoP is rurality. Although Japan has several rural areas, the SoP in rural areas and the effect of rurality on SoP have not been investigated. This study aimed to describe SoP in Japanese primary care settings and examine the association between rurality and SoP.

**Methods:**

This cross-sectional study included PCPs in Japan. The participants were randomly sampled from the mailing list of the Japan Primary Care Association. The Scope of Practice Inventory (SPI) and Scope of Practice for Primary Care (SP4PC) were used as indicators of SoP. The Rurality Index for Japan (RIJ) was used for rurality. This study compared the number of items of SPI (total score, inpatient care, urgent care and ambulatory care) and SP4PC experienced by > 80% of all PCPs in the most urban (RIJ:1–10) and rural areas (RIJ: 91–100). A multivariable linear regression analysis was also performed to examine the relationship between the RIJ and SPI/SP4PC.

**Results:**

Of 1,000 potential participants, 299 physicians responded to the survey (response rate: 29.9%). PCPs in the most rural areas experienced a greater number of items in the inpatientl/urgent care domains of the SPI and SP4PC than those in the most urban areas. The RIJ was the only common factor for a broader SoP in both the SPI and SP4C models. The coefficients of SoP were 0.09 (95% confidence interval: 0.03–0.16) in the SPI model and 0.017 (0.005–0.03) in the SP4PC model.

**Conclusion:**

Rurality was considerably associated with SoP. The findings of this study will be helpful in understanding the SoP on rural and urban areas.

**Supplementary Information:**

The online version contains supplementary material available at 10.1186/s12875-023-02250-y.

## Introduction

Comprehensive care with a broad scope of practice (SoP) is a core domain of family medicine [1]. Although there is no universal consensus on the definition of “broad” or “full” SoP, comprehensive medical care by the College of Family Physicians of Canada includes primary care, emergency care, home and long-term care, hospital care, and maternal and newborn care [2]. Providing a broad SoP is associated with lower admission rates and healthcare costs [3]. A broader SoP can provide patients with the “right care, at the right time, in the right place” [3]. As a result, a broader SoP is associated with efficient care by avoiding costly care [3]. Moreover, a broader SoP is related to a lower burnout incidence among physicians [4, 5]. The environment for practicing desirable SoP for individual physicians may be important in preventing burnout [5, 6]. In addition to individual factors, system factors also play a key role in maintaining a broader SoP. Rural PCPs are required to have a broader SoP [5, 6].

The SoP is determined by various factors. A qualitative study by Russel et al. identified four domains influencing the SoP of primary care physicians based on focus group interviews: personal, workplace, environment, and population factors [7]. Of them, “environment”, especially geography, is the most influential factor for SoP in several countries [8, 9]. This is because of limited access to specialists [8].

Although Japan has several rural areas, including remote islands [10], the SoP in rural areas and how rurality affects it have not yet been investigated. Thus, this study aimed to describe the SoP in Japanese primary care settings and examine the association between rurality and the SoP. We hypothesized that Japanese primary care physicians (PCPs) in rural areas have a broader SoP than their counterparts in urban areas. The results would provide basic information to understand the actual and required SoP based on rurality in Japanese primary care settings.

## Methods

### Study design

A cross-sectional study using an online survey.

### Setting and participants

The participants were physicians at the Japan Primary Care Association. This study used a mailing list from the association. The mailing list includes 4,147 members as of December 2022. Of them, 3,317 were PCPs. We offered a 500 JPY Amazon gift card to the participants as an incentive. The study recruited 1,000 potential participants using a simple random sampling because the budget for the incentive was limited. The questionnaires were distributed to members of the mailing list. We excluded non-physician members such as nurses or pharmacists.

### Measures

#### Outcome variable

The Scope of Practice Inventory (SPI) [9] and the Scope of Practice for Primary Care (SP4PC) [11] as indicators of SoP.

We employed the SPI which was developed in 2015 in Japan [9]. SPI is composed of three domains and 68 questions: the domains are inpatient care, urgent care, and ambulatory care [9]. The examples of the items are as follows: “Performing thoracocentesis: yes/no,” “Initial management of febrile seizure; yes/no,” and “Diagnosing and managing diabetes Diagnosing: yes/no” [9]. The SPI showed good reliability and validity in the Japanese setting. The SPI scores ranged from 0 to 68 [9].

The SP4PC was also used in this study. The SP4PC includes 22 questions, and the score ranges from 0 to 30 [11]. SP4PC is composed of emergency care, geriatric medicine, adult medicine, care for children, deliveries, preoperative care etc. [11]. The items of SPI and SP4PC are shown in Supplementary Table 1. Both scores of SPI and SP4PC were calculated by simply adding each item. The score of total SPI includes the score of inpatient care, urgent care and ambulatory care.

#### Exposure variable

The Rurality Index for Japan, an indicator of rurality [12].

The Rurality Index for Japan (RIJ) is composed of four factors: population density, distance to secondary or tertiary care hospitals, remote islands, and heavy snow areas [12]. The final score was calculated by adding each factor with weight [12]. The index describes rurality from 1 to 100 (100 means most rural) [12].

### Covariates

Based on previous literature [8], this study adjusted for sex, years of clinical experience, clinical setting (clinic or hospital), certification status, and experience of practice in rural areas as confounding factors. These variables were obtained through an online self-administered survey.

Sex: a categorical variable (male, female, others).

Years of clinical experience: a continuous variable.

Clinical setting: a categorical variable (clinic, hospital < 199 beds, hospital ≥ 200 beds, others).

Having certification: A binary variable. The certification indicates “certified family physician” and “certified primary care physicians”, which is approved by the Japan Primary Care Association.

Experience of rural practice: A binary variable. In this question, “rural” was based on the subjective perception of the participant.

### Statistical analyses

Categorical variables are presented as numbers and proportions. Continuous variables are described as medians and interquartile ranges. The relationship between the RIJ and SPI/SP4PC was visualized using box plots. To demonstrate the difference in SoP between urban and rural areas, we described the SoP experienced by > 80% of PCPs between the most urban (RIJ: 1–10) and rural areas (RIJ: 91–100). We listed each item of SPI and SP4PC which were conducted by 80% of PCPs in the most urban and rural areas. We also performed a multivariable linear regression analysis to examine the relationship between the RIJ and SPI (total and each domain)/SP4PC. The confounding factors described above were also included in the model. There was no missing data in the study. For sensitivity analysis, we conducted the analysis including participants who only worked in a clinic.. We also analyzed the participants, excluding trainees within 2 years of completion of medical school as the SoP would depend on their rotation in the first two years of training [13]. All statistical analyses were conducted using Stata Statistical Software: Release 15 (College Station, TX, StataCorp, LLC).

## Results

**Fig. 1:**
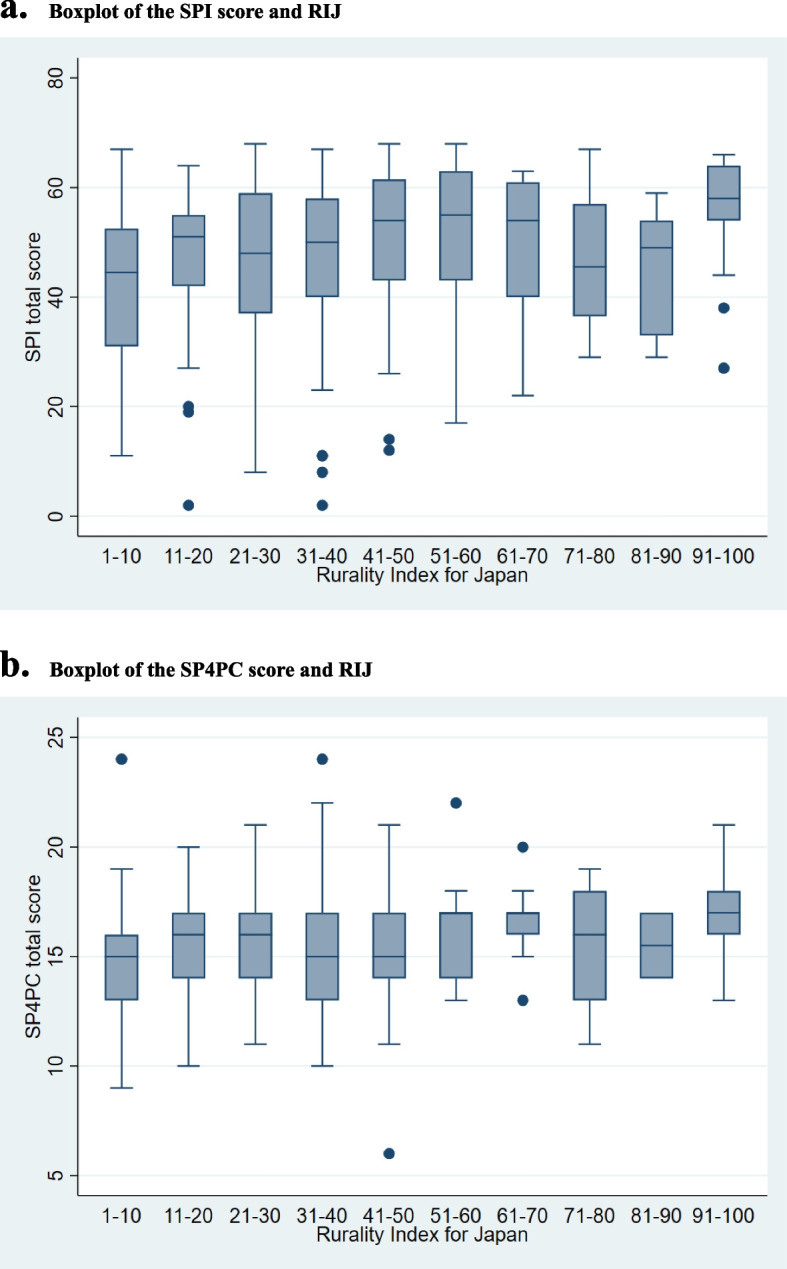
**a** Boxplot of the SPI score and the RIJ. **b** Boxplot of the SP4PC score and the RIJ. SPI: Scope of Practice Inventory, SP4PC, Scope of Practice for Primary Care: RIJ: Rurality Index for Japan

Of the 1,000 potential participants, 299 physicians responded to the survey (response rate, 29.9%). We excluded eight participants because their ages were < 10 years or > 300 years and decided that these were input errors. Ultimately, 291 physicians were included in the analysis. Table 1 presents the characteristics of the participants. Supplementary Figure S1a and b show histograms of the SPI and SP4PC of the participants. The boxplots of SPI/SP4PC and RIJ are shown in Fig. 1a and b. The SoP score of the most rural group was higher than that of the most urban group.

**Table 1:** Characteristics of the participants (*n* = 291)

Sex	
Male	225 (77.3)
Female	66 (22.7)
Others	0 (0)
Age	
20–29	21 (7.2)
30–39	69 (23.7)
40–49	99 (34.0)
50–59	57 (19.6)
60–69	42 (14.4)
70–79	1 (0.3)
80–89	2 (0.7)
Years of clinical experience	
1–9	76 (26.1)
10–19	82 (28.2)
20–29	66 (22.7)
30–39	50 (17.2)
40–49	14 (4.8)
50–59	3 (1.0)
Clinical setting	
Clinic	126 (43.3)
Hospital < 199 beds	72 (24.7)
Hospital ≥ 200 beds	90 (30.9)
Others	3 (1.0)
Having certification of family physician/primary care physician	
Yes	204 (70.1)
No	87 (29.9)
Experience of rural practice	
Yes	189 (64.9)
No	102 (35.1)
RIJ	29 (12–42)
SoP	
SPI (max: 68)	50 (38–58)
Inpatient care (max: 25)	20 (13–24)
Urgent care (max: 27)	15 (7–22)
Ambulatory care (max: 16)	16 (15–16)
SP4PC (max: 30)	15 (14–17)

Categorical variables are described as numbers and proportions. Continuous variables are described as medians and interquartile ranges

**Fig. 2:**
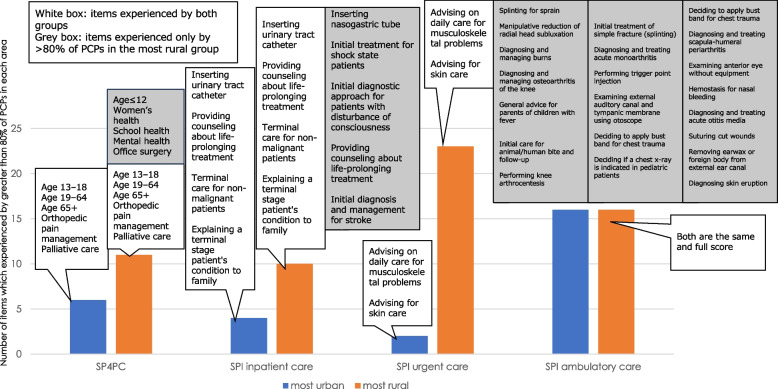
Scope of practice experienced by greater than 80% of all physicians in the most urban and rural areas

Figure 2 shows the difference in the SoP experienced by > 80% of PCPs between the most urban and rural areas. Although both groups, the most urban and the most rural, experienced similar items in the ambulatory care domain of the SPI, PCPs in the most rural area experienced a broader SoP in other domains of the SPI and SP4PC, especially in the urgent care domain of the SPI. The association between the SoP described by SPI/SP4PC and each variable is shown in Table 2. In the model using SPI, female sex (coefficient -4.36, 95% confidence interval: -8.36 to -0.36), years of clinical experience (-0.22, -0.37 to -0.08), and working outside a clinic or hospital (-27.36, -43.28 to -11.44) were associated with a narrower SoP. In this model, hospitals with < 199 beds (6.81, 2.75 to 10.87) and RIJ (0.09, 0.03 to 0.16) had a broader SoP. In the SP4PC model, years of clinical experience (-0.03, -0.06 to -0.001) and working outside a clinic or hospital (-3.10, -6.19 to -0.004) were associated with a narrower SoP, and RIJ (0.02, 0.01 to 0.03) was the only statistically significant factor for a broader SoP. Regarding the subdomains of SPI, the coefficients of RIJ were (0.01 -0.02 to 0.04) in inpatient care, (0.08, 0.04 to 0.11) in urgent care and (-0.001, -0.01 to 0.01) in ambulatory care, respectively. As a sensitivity analysis, we only included the participants who worked at clinic (*N* = 126). The coefficients of RIJ were (0.08, -0.002 to 0.17) in SPI total score and (0.02, 0.004 to 0.03) in SP4PC, respectively. Also, we conducted the analysis which excluded trainees (*N* = 278): the coefficients of RIJ were (0.09, 0.02 to 0.16) in SPI total score and (0.02, 0.01 to 0.03) inSP4PC. In the sensitivity analyses, we also adjusted for sex, years of clinical experience, clinical setting, certification status, and experience in rural practice. The sensitivity analyses indicated similar trends for the SoP.

**Table 2:** Association between the SoP described by SPI/SP4PC and each variable: the results of multivariable linear regression

Variables	Coefficient (95% CI, *p*-value)
SoP described by SPI	
Sex	
Male	Reference
Female	-4.36 (-8.36 to -0.36, *p* = 0.033)
Years of clinical experience	-0.22 (-0.37 to -0.08, *p* = 0.003)
Clinical setting	
Clinic	Reference
Hospital < 199 beds	6.81 (2.75 to 10.87, *p* = 0.001)
Hospital ≥ 200 beds	-0.31 (-4.14 to 3.52, *p* = 0.872)
Others	-27.36 (-43.28 to -11.44, *p* = 0.001)
Having certification	-0.65 (-4.29 to 2.99, *p* = 0.725)
Experience of rural practice	2.61 (-0.88 to 6.09, 0.142)
RIJ	0.09 (0.03 to 0.16, *p* = 0.007)
SoP described by SP4PC	
Sex	
Male	Reference
Female	-0.54 (-1.32 to 0.24, *p* = 0.171)
Years of clinical experience	-0.03 (-0.06 to -0.001, *p* = 0.046)
Clinical setting	
Clinic	reference
Hospital < 199 beds	0.27 (-0.52 to 1.06, *p* = 0.503)
Hospital ≥ 200 beds	-0.54 (-1.28 to 0.21, *p* = 0.157)
Others	-3.10 (-6.19 to -0.004, *p* = 0.05)
Having certification	0.13 (-0.57 to 0.84, *p* = 0.71)
Experience of rural practice	0.28 (-0.40 to 0.96, *p* = 0.419)
RIJ	0.02 (0.005 to 0.03, *p* = 0.008)

## Discussion

This study described the SoP of PCPs in Japan and compared the SoP in the most urban and rural areas. This study also revealed that high rurality was associated with a broad SoP. The results are helpful in understanding the actual and required SoP for working in rural areas.

In Japan, there are limited studies about SoP. The previous research about the development of SPI in Japan only described SoP in Japan with a validated measure. The previous study revealed the mean of total SPI: 36 (standard deviation 15.67), inpatient care 11.93 (8.31), urgent care 9.85 (8.09), and ambulatory care 14.22 (2.88) [9]. In the study, the mean of the total and each domain of SPI were 46.7 (14.71), inpatient care: 17.52 (7.247), urgent care: 14.38 (8.57), and ambulatory care: 14.38 (8.57). The participants of the study covered a broader SoP compared with that in the previous study [9]. This can be explained by the higher rate of rural practice experience in the present study (64.9%) compared with that in the previous study (29.9%) [9]. In addition, 62.1% of the participants in the previous study practiced in urban areas [9]. They could affect the difference between the previous and present study.

A higher rurality score was associated with a broader SoP. In addition, female sex and longer years of clinical experience were associated with a narrower SoP, and the results were similar to those of previous studies in Japan and other countries [8, 9, 14, 15]. Regarding rurality and SoP, a possible explanation is that limited access to specialist care may contribute to a broader SoP [7]. Additionally, a shortage of other health professionals in rural areas may be associated with a broad SoP of PCPs [16]. The shortage of specialists and other health care providers such as nurses in rural areas also exists in Japan. This can be the reason that a broader SoP is needed for rural PCPs in Japan [17–20]. The direction of the trend was similar in sensitivity analyses. In Japan, trainees within 2 years of completion of medical school need to rotate internal medicine, surgery, pediatrics, obstetrics and gynecology, and psychiatry [21]. Their SoP may depend on the phase of their rotation. Therefore, we excluded the trainees. The results were similar to the main analysis. One of the reasons may be that the number of trainees in the study was small (13) and did not affect the results. Also, the SoP of the trainees might be narrow compared to that of physicians who completed the training. Thus, the sensitivity analysis might describe the actual SoP of the Japanese PCPs. In this study, the SPI urgent score had a higher coefficient than that of other domains such as inpatient and ambulatory care. This can be explained by the fact that the SPI urgent care domain includes care for minor emergencies and care for children. These procedures may need to be covered by PCPs in areas where access to specialists is limited. This indicated that a skill set for urgent care is required for PCPs in rural areas compared with those in urban areas. A previous study in Canada reported that rural PCPs tended to engage in emergency care more than PCPs in urban areas [22]. The results also showed that the difference between the most urban and rural areas is evident in the SPI urgent score. In terms of the sensitivity analysis which targeted the participants working at a clinic, SP4PC was significantly associated with the RIJ. Although SPI had a similar trend, the results were not statistically significant. The possible explanation was that one of the three subdomains of SPI was inpatient care. SP4PC only included 3 inpatient-care-related items of all 22 items: inpatient care, pre-operative care, and post-operative care. Thus, SPI was relatively likely to be influenced by the SoP of participants working in a hospital.

In terms of other factors, female sex was associated with a narrower SoP. This can be explained by that female physicians tend to work on a part-time basis [23]. Moreover, in Japan, approximately 30% of all female physicians have career breaks during graduation 10–15 years for childbearing etc. [24]. The fact also can be related to a narrower SoP. For years of clinical experience, the previous literature reported older physicians are less likely to touch acute or emergency care and house calls [25, 26]. These characteristics of practice may influence the results.

### Clinical implication

The study provide insight into the SoP in most rural areas as it described the differences between SoP in most urban and rural areas. This study provides important information regarding the required SoP and training for working in most rural areas of Japan. As a broader SoP may be related to the prevention of physician burnout, working in rural areas could mitigate this risk [5].

### Strengths of the study

This is the first study to examine RIJ and SoP. In addition, the study compared the SoP in most urban and rural areas. As “rural” does not mean uniform [27], the required SoP may vary by the degree of rurality and context. Describing the SoP based on rurality provides important information. Another strength of this study is that the participants were randomly selected from members of the largest academic society of PCPs in Japan.

### Limitations of the study

This study had some limitations. First, because of the nature of the cross-sectional study, a causal relationship between the RIJ and SoP could not be demonstrated. However, acquiring a broader SoP usually requires training in rural areas in Japan. In addition, a broader SoP could not be a reason for working in rural areas. Therefore, these results cannot be explained by reverse causality. Second, the response rate was relatively low. Therefore, potential participants with a broader SoP might have taken part in the survey. Thus, our results might have overestimated the SoP of PCPs in Japan. These results need to be carefully extrapolated to PCPs in overall Japan.

## Conclusions

This is the first study to examine the relationship between rurality and SoP. Rurality is considerably associated with SoP. The findings of this study will be helpful in understanding the SoP on rural and urban areas.

### Supplementary Information

**Additional file 1: Supplementary Table 1.** Items of the Scope of Practice for Primary Care (SP4PC) [11].

**Additional file 2: Figure S1.** a. Histogram of the SPI score of the participants. b. Histogram of the SP4PC score of the participants.

## Data Availability

The datasets generated and analyzed in the current study are not publicly available as we did not receive written informed consent for data sharing.

## Abbreviations

PCP: Primary care physician
RIJ: Rurality Index for Japan
SoP: Scope of Practice
SP4PC: Scope of Practice for Primary Care
SPI: Scope of Practice Inventory

## References

[1] Starfield B, Shi L, Macinko J (2005). Contribution of primary care to health systems and health. Milbank Q.

[2] Aggarwal M, Oandasan I. Scope of Practice of family physicians in Canada: an outcomes of training project evidence summary. 2022. https://www.cfpc.ca/futurefp. Accessed Jan 2022.

[3] Bazemore A, Petterson S, Peterson LE, Phillips RL (2015). More comprehensive care among family physicians is associated with lower costs and fewer hospitalizations. Ann Fam Med.

[4] Zomahoun HTV, Samson I, Sawadogo J, Massougbodji J, Gogovor A, Diendéré E, Turgeon F (2021). Effects of the scope of practice on family physicians: a systematic review. BMC Fam Pract.

[5] Weidner AKH, Phillips RL, Fang B, Peterson LE (2018). Burnout and scope of practice in new family physicians. Ann Fam Med.

[6] Coutinho AJ, Cochrane A, Stelter K, Phillips RL, Peterson LE (2015). Comparison of intended scope of practice for family medicine residents with reported scope of practice among practicing family physicians. JAMA.

[7] Russell A, Fromewick J, Macdonald B, Kimmel S, Franke K, Leach K (2021). Drivers of scope of practice in family medicine: a conceptual model. Ann Fam Med.

[8] Wong E, Stewart M (2010). Predicting the scope of practice of family physicians. Can Fam Physician.

[9] Ie K, Ichikawa S, Takemura YC (2015). Development of a questionnaire to measure primary care physicians’ scope of practice. BMC Fam Pract.

[10] Health Policy Unit, Graduate School of Public Policy, Tokyo University. Rural Health. 2023. https://warp.ndl.go.jp/collections/info:ndljp/pid/9976637/www.pp.u-tokyo.ac.jp/HPU/seminar/2014-10-12/d/Guideline_F19_rev.pdf. Accessed 24 Jul 2023.

[11] O’Neill T, Peabody MR, Blackburn BE, Peterson LE (2014). Creating the individual scope of practice (I-SOP) scale. J Appl Meas.

[12] Kaneko M, Ikeda T, Inoue M, Sugiyama K, Saito M, Ohta R (2023). Development and validation of a rurality index for healthcare research in Japan: a modified Delphi study. BMJ Open.

[13] Kato D, Ryu H, Matsumoto T, Abe K, Kaneko M, Ko M (2019). Building primary care in Japan: literature review. J Gen Fam Med.

[14] Hutten-Czapski P, Pitblado R, Slade S (2004). Short report: scope of family practice in rural and urban settings. Can Fam Physician.

[15] Ward ZD, Morgan ZJ, Peterson LE (2021). Family physician burnout does not differ with rurality. J Rural Heal.

[16] Nasim U, Morgan ZJ, Peterson LE (2021). The declining scope of practice of family physicians is limited to urban areas. J Rural Heal.

[17] Matsuzawa Y, Konishi M, Nakai M, Saigusa Y, Taguri M, Gohbara M (2020). In-hospital mortality in acute myocardial infarction according to population density and primary angioplasty procedures volume. Circ J.

[18] Yoshida S, Matsumoto M, Kashima S, Koike S, Tazuma S, Maeda T (2019). Geographical distribution of family physicians in Japan: a nationwide cross-sectional study. BMC Fam Pract.

[19] Matsumoto M, Kimura K, Inoue K, Kashima S, Koike S, Tazuma S (2018). Aging of hospital physicians in rural Japan: a longitudinal study based on national census data. PLoS One.

[20] The Ministry of Health, Labour and Welfare. Maintain sufficient manpower to support social security system. https://www.mhlw.go.jp/english/wp/wp-hw2022/dl/summary.pdf. Accessed 9 Dec 2023.

[21] Muroya S, Ohde S, Takahashi O, Jacobs J, Fukui T (2021). Differences in clinical knowledge levels between residents in two post-graduate rotation programmes in Japan. BMC Med Educ.

[22] Wenghofer EF, Kam SM, Timony P, Strasser R, Sutinen J (2018). Geographic variation in FP and GP scope of practice in Ontario. Can Fam Physician.

[23] Boerma WG, van den Brink-Muinen A (2000). Gender-related differences in the organization and provision of services among general practitioners in Europe: a signal to health care planners. Med Care.

[24] The Ministry of Health, Labour and Welfare. Report of the study group on the supply and demand of physicians. http://www.mhlw.go.jp/shingi/2006/07/dl/s0728–9c.pdf. Accessed 9 Dec 2023.

[25] Chan BT (2002). The declining comprehensiveness of primary care. CMAJ.

[26] Chan B, Anderson GM, Thériault ME (1998). Patterns of practice among older physicians in Ontario. CMAJ.

[27] Wenghofer EF, Timony PE, Gauthier NJ (2014). “Rural” doesn’t mean “uniform”: northern vs southern rural family physicians’ workload and practice structures in Ontario. Rural Remote Health.

