# Supplementary material for: Primary care physicians working in rural areas provide a broader scope of practice: a cross-sectional study
Source: BMC Prim Care. 2024 Jan 2;25:9. doi: 10.1186/s12875-023-02250-y (PMC10759475; doi:10.1186/s12875-023-02250-y)
Supplement: Supplementary file 1 — Additional file 1: Supplementary Table 1. Items of the Scope of Practice for Primary Care (SP4PC) [11]. [file 12875_2023_2250_MOESM1_ESM.docx]

**Supplementary Table 1**

Items of the Scope of Practice for Primary Care (SP4PC)^11^

| Please answer the following questions (yes/no) |
| --- |
| Do you see the patients who are:  Age ≤12?  Age 13–18?  Age 19–64?  Age 65+? |
| Do you provide the following care in your practice? (yes/no) |
| Inpatient care  Emergent care  Urgent care  Pain management  Palliative care  Office surgery  Major surgery  Pre-operative care  Post-operative care  Sports medicine  Occupation/Industrial medicine  Mental health  Orthopedic problems  Women’s health  School health  Obstetrical delivery  Prenatal care  Newborn care |
