# Supplementary material for: Primary care physicians working in rural areas provide a broader scope of practice: a cross-sectional study
Source: BMC Prim Care. 2024 Jan 2;25:9. doi: 10.1186/s12875-023-02250-y (PMC10759475; doi:10.1186/s12875-023-02250-y)

**Figure S1a. Histogram of the SPI score of the participants**


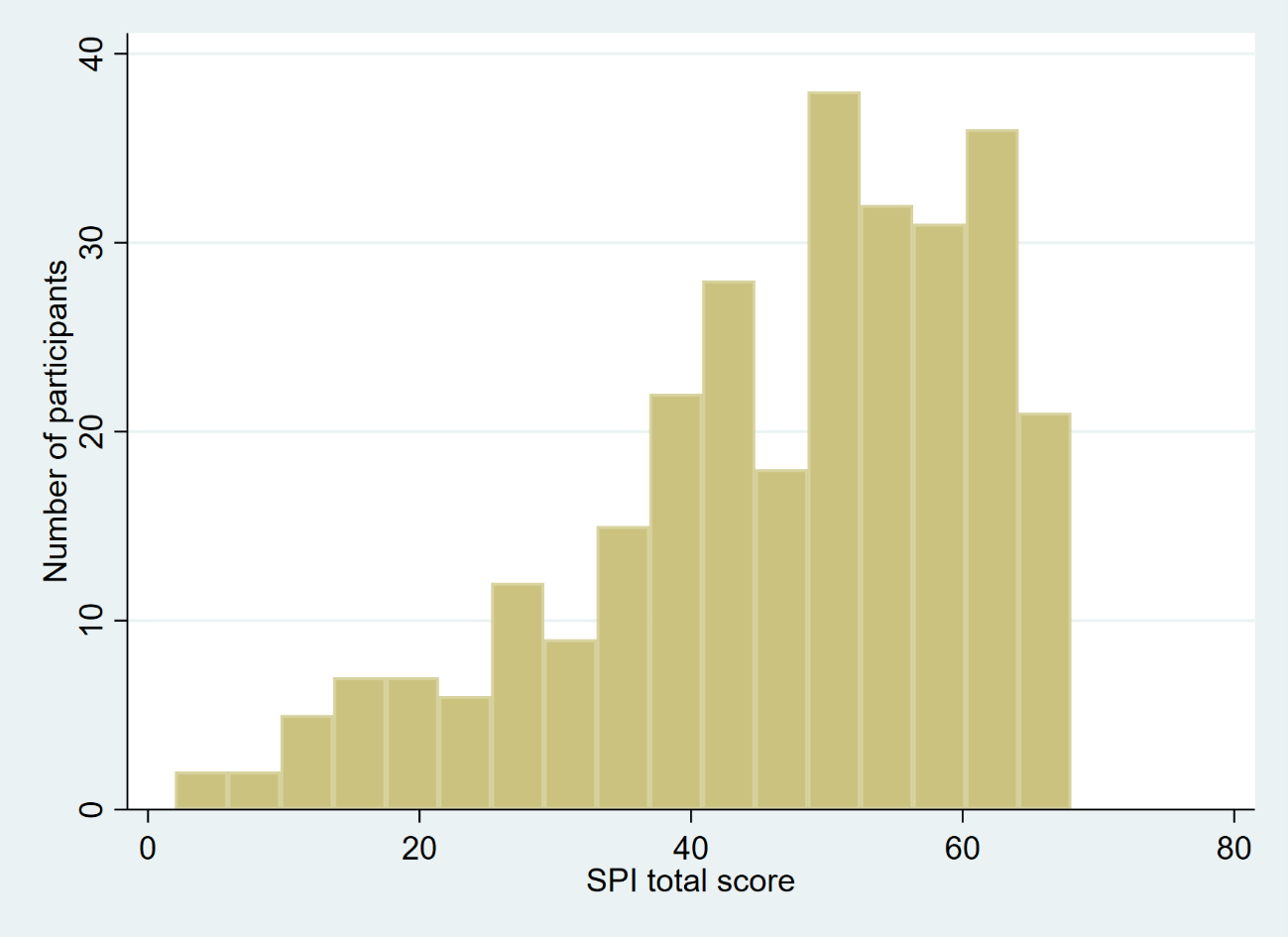


**Figure S1b. Histogram of the SP4PC score of the participants**


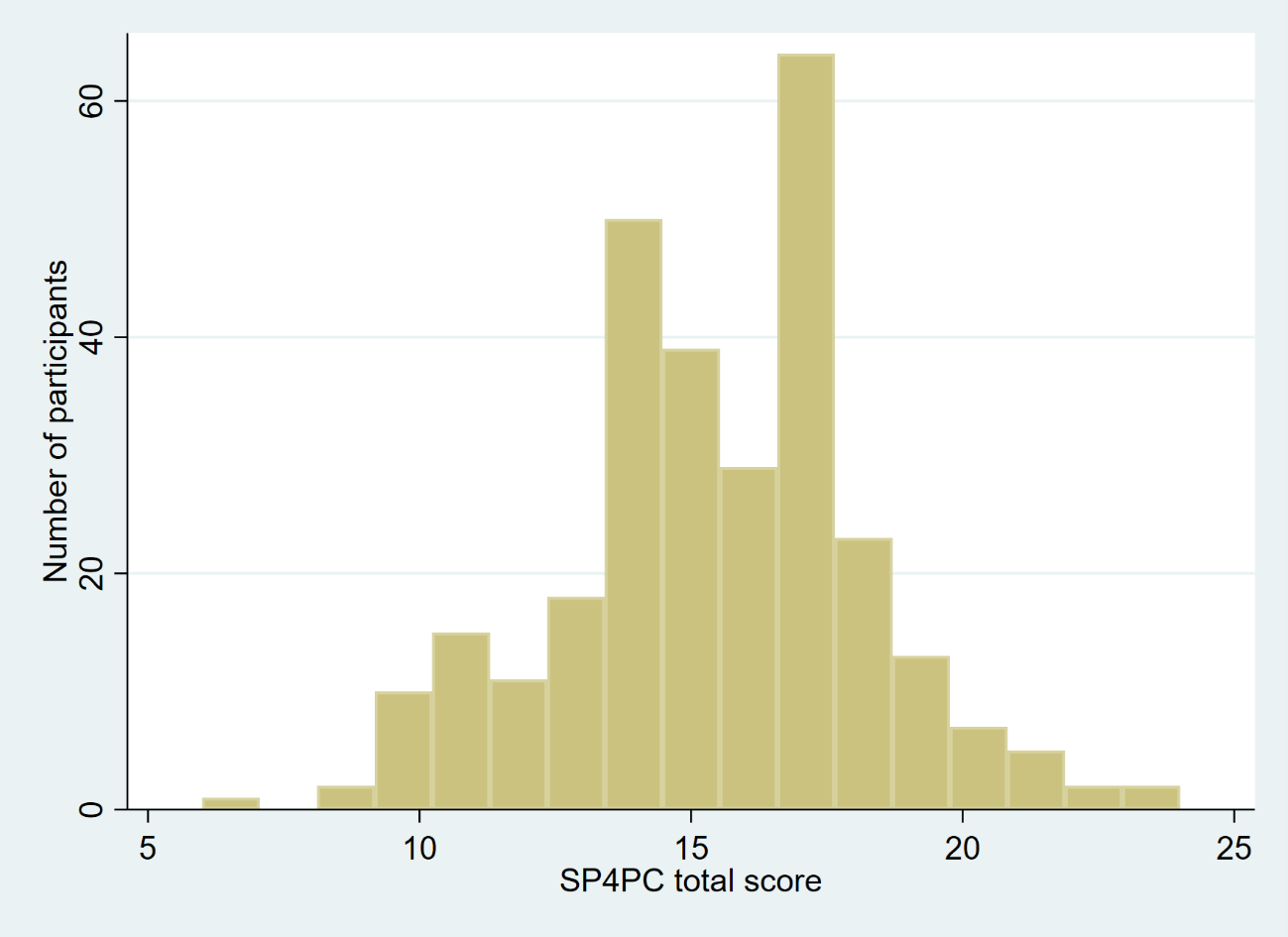

Supplement: Supplementary file 2 — Additional file 2: Figure S1. a. Histogram of the SPI score of the participants. b. Histogram of the SP4PC score of the participants. [file 12875_2023_2250_MOESM2_ESM.docx]
